# Using comparative genomic hybridization to survey genomic sequence divergence across species: a proof-of-concept from *Drosophila*

**DOI:** 10.1186/1471-2164-11-271

**Published:** 2010-04-29

**Authors:** Suzy CP Renn, Heather E Machado, Albyn Jones, Kosha Soneji, Rob J Kulathinal, Hans A Hofmann

**Affiliations:** 1 Department of Biology, Reed College, Portland, OR 97202, USA; 2 Department of Mathematics, Reed College, Portland, OR 97202, USA; 3 Boston University School of Medicine, Boston MA 02118, USA; 4 Department of Biology, Temple University, Philadelphia, PA, 19122, USA; 5 Section of Integrative Biology, Institute for Molecular and Cellular Biology, Institute for Neuroscience, The University of Texas at Austin, Austin, TX, 78712, USA

## Abstract

**Background:**

Genome-wide analysis of sequence divergence among species offers profound insights into the evolutionary processes that shape lineages. When full-genome sequencing is not feasible for a broad comparative study, we propose the use of array-based comparative genomic hybridization (aCGH) in order to identify orthologous genes with high sequence divergence. Here we discuss experimental design, statistical power, success rate, sources of variation and potential confounding factors. We used a spotted PCR product microarray platform from *Drosophila melanogaster *to assess sequence divergence on a gene-by-gene basis in three fully sequenced heterologous species (*D. sechellia*, *D. simulans*, and *D. yakuba*). Because complete genome assemblies are available for these species this study presents a powerful test for the use of aCGH as a tool to measure sequence divergence.

**Results:**

We found a consistent and linear relationship between hybridization ratio and sequence divergence of the sample to the platform species. At higher levels of sequence divergence (< 92% sequence identity to *D. melanogaster*) ~84% of features had significantly less hybridization to the array in the heterologous species than the platform species, and thus could be identified as "diverged". At lower levels of divergence (≥ 97% identity), only 13% of genes were identified as diverged. While ~40% of the variation in hybridization ratio can be accounted for by variation in sequence identity of the heterologous sample relative to *D. melanogaster*, other individual characteristics of the DNA sequences, such as GC content, also contribute to variation in hybridization ratio, as does technical variation.

**Conclusions:**

Here we demonstrate that aCGH can accurately be used as a proxy to estimate genome-wide divergence, thus providing an efficient way to evaluate how evolutionary processes and genomic architecture can shape species diversity in non-model systems. Given the increased number of species for which microarray platforms are available, comparative studies can be conducted for many interesting lineages in order to identify highly diverged genes that may be the target of natural selection.

## Background

Comparison of genomic DNA sequence among closely related strains or species is a powerful approach with which to identify heterogeneity in evolutionary processes such as selection, mutation rates, and rates of introgression, as well as to unmask phylogenetic relationships. However, even with the recent advances in DNA sequencing technology and rapidly dropping costs, complete genome sequence data are not readily available for many closely related eukaryotes that serve as model systems for organismal evolution [but see [1,2]]. As an alternative, comparative genomic hybridization (CGH) offers a means to estimate sequence divergence.

Although the use of genomic DNA (gDNA) hybridization for phylogenetic analyses and genome-wide estimation of sequence similarity date to long before vast amounts of sequence data became available [e.g. [3,4]], this approach has experienced a renaissance with the development of genomic tools, specifically microarrays. On a relatively coarse level, array-based CGH (aCGH) has been widely applied to identify chromosomal aberrations underlying cancer [for review see [5]]. When gDNA isolated from a tumor is competitively hybridized against gDNA isolated from normal tissue, genomic regions that have been deleted in the genome of the tumor cells will fail to hybridize to the array features while genomic regions that have been duplicated (amplified) in the genome of the tumor cells will hybridize at a ratio of 2:1 (or greater). At a finer level of resolution, modifications of this technique have allowed microarray-based genotyping of single nucleotide polymorphisms within and between populations [e.g. *Arabidopsis*: [6], stickleback fish: [7]]. Array-based techniques can also be applied to genome-scale comparisons between closely related species (or strains) in order to conduct a (nearly) complete analysis of sequence divergence on a gene-by-gene basis.

Unlike microarrays designed for genotyping known polymorphisms [reviewed by [8]] or re-sequencing [human: [9], *Arabidopsis*: [10]], microarrays designed for gene expression studies can also be used to compare the genomic content (in coding sequence) of closely related species. In a typical experiment, gDNA from the platform species (from which the microarray was constructed) is compared on the array to gDNA from another (heterologous) species of interest. This technique has been used to reveal genomic regions likely involved in an organism's ability to inhabit a specific environment [*Chlamydia trachomatis *tissue specificity: [11], *Sinorhizobium meliloti *root symbiont: [12], *Clostridium difficile *host specificity: [13]], pathogenicity [*Yersinia pesits*: [14,15], *Mycobacterium tuberculosis*: [16], *Vibrio cholerae*: [17]], genomic duplications and deletions associated with population divergence and speciation [*Anopheles gambiae*: [18,19]], and genomic regions that differentiate humans from other primate species [20,21]. While most studies rely only on presence or absence metrics, a few studies have suggested that the relationship between hybridization signal ratio using aCGH and nucleotide identity is roughly log-linear [11,22]. Using this relatively inexpensive approach, it is possible to identify rapidly evolving genes [*Paxillus involutus*: [23]] and in some cases lend insight to phylogenetic relationships [*Shewanella*: [24], *Salmonella*: [25], *Saccharomyces*: [26]]. While the majority of these examples derive from studies in microbes, the technique is amenable to genomes of any size. It must be noted, of course, that array-based comparisons do not reveal the actual genomic sequence for the novel genes of interest. Instead, an estimate of sequence identity is obtained at a price and effort far below that of whole genome sequencing.

In the present study, we examine the relationship between hybridization ratio and sequence divergence using a cDNA microarray constructed for *D. melanogaster*. The availability of complete genome assemblies [27] for *Drosophila melanogaster *as well as three other Drosophilid species, *D. simulans*, *D. sechellia *(both 2-3 MY diverged relative to *D. melanogaster*) and *D. yakuba *(10-15 MY diverged relative to *D. melanogaster*)[28] provides us with a unique opportunity to demonstrate the degree to which hybridization ratio reflects underlying sequence divergence. It is not our goal to devise an explicit model to explain variation in hybridization due to other sequence characteristics; rather, we demonstrate success rate, discuss the effects of a few characteristics contributing to variation in hybridization kinetics, and provide an example for the use of aCGH that can be applied to non-model organisms.

We show that sequence divergence between orthologous genes can be successfully detected for closely and not so closely related species. Approximately 40% of the variation in gDNA hybridization ratios can be explained by sequence divergence, as measured by nucleotide dissimilarity between sequences. Other sequence-specific characteristics also explain part of the variation in hybridization ratio, and become more prominent with increased sequence divergence. Similarly, technical variation increases with increasing sequence divergence; however, this last source of variation can be overcome with increased replication. We demonstrate the potential for functional analysis and the generation of testable hypotheses based upon hybridization statistics for Gene Ontology annotation.

## Results and Discussion

### Detection of reduced hybridization

In order to identify array features for which hybridization strength was reduced in each of three heterologous Drosophilid species, two direct comparisons to *D. melanogaster *were performed. After filtering for unusable array features (low quality or intensity), approximately 80% of the array features were available for analysis in each species. From these data we identified array features for which the genomic DNA hybridization signal for each species was reduced compared with *D. melanogaster*. As predicted by their divergence time relative to *D. melanogaster*, the fraction of array features that showed a statistically significant reduction in genomic hybridization signal relative to *D. melanogaster *was similar for *D. sechellia *(45.4%) and *D. simulans *(55.8%), and was considerably greater for the more distant *D. yakuba *(70.6%) (P < 0.1 FDR corrected) (Table 1). This result, a first for this degree of divergence among multicellular organisms with complex genomes, is consistent with that obtained by Edwards-Ingram et al. [26]. These authors showed that the "molecular taxonomy" of yeast (*Saccharomyces sensu stricto*) as determined by aCGH using a binary presence-absence and parsimony-based method closely matched the phylogeny inferred from the complete genome sequences [29]. Similarly, the neighbor-joining and parsimony-based trees constructed with aCGH data from different *Salmonella *subtypes [25] correspond with the phylogenetic hypotheses inferred from genomic sequence [30].

**Table 1:** Features identified as diverged from *D. melanogaster*.

H. Species	# Analyzed	P < 0.1 FDR	P < 0.05 FDR	P < 0.01 FDR
*D. sechellia*	18374	45%	38%	23%
*D. simulans*	16325	56%	34%	21%
*D. yakuba*	17724	71%	66%	58%

H. Species: the heterologous species used in the 2-array comparison with *D. melanogaster*.

### Detection of sequence divergence

**Figure 1:**
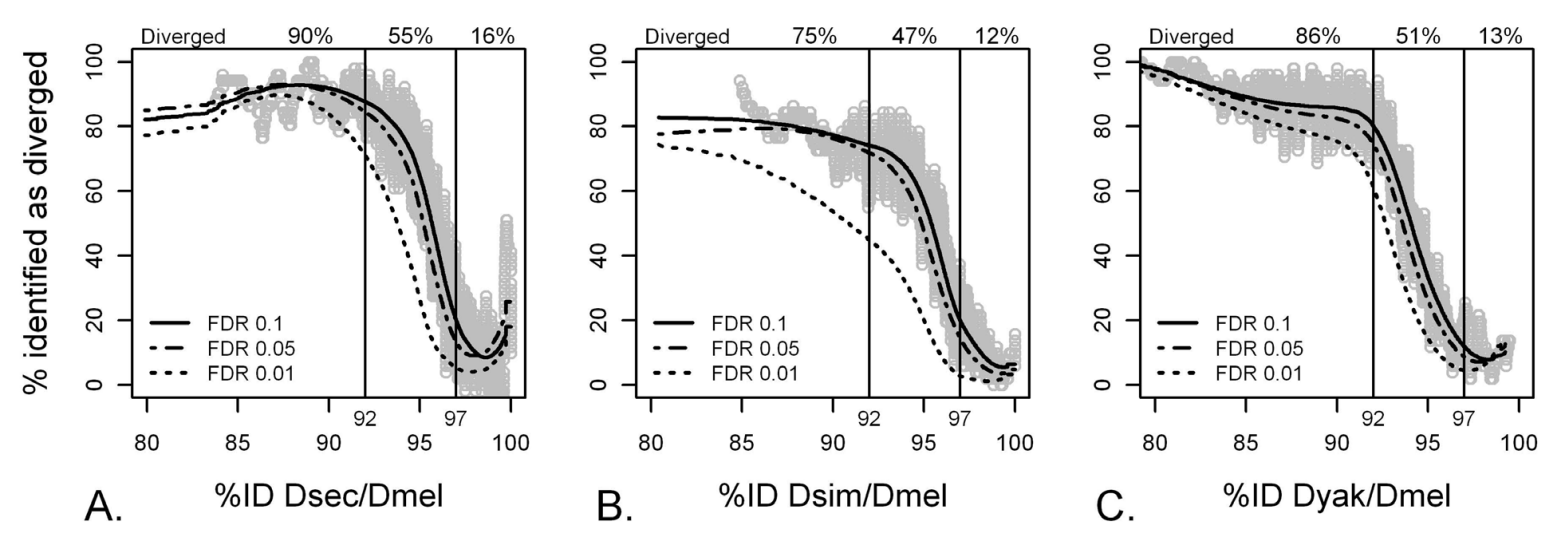
**Identification of sequence divergence by aCGH**. *D. melanogaster *compared with *D. sechellia *(A), *D. simulans *(B) and *D. yakuba *(C). The percent of array features that were identified as "diverged" based on statistical analysis of hybridization ratio (y-axis) is reported as function of actual sequence divergence (x-axis). Grey points indicate the percentage for a moving window of 51 array features at P < 0.1, corrected for false discovery. Lowess-smoothed curves summarize these values for P < 0.1 FDR (solid), P < 0.05 FDR (dashed) and P < 0.01 FDR (dotted).

In order to test the ability to detect highly diverged sequences with aCGH, we used BLAST to query the full genome assemblies of each of the heterologous species with the predicted *D. melanogaster *probe sequences to provide a measure of sequence divergence for comparison to the array-based measures. The percent nucleotide similarity of the top BLAST hit for the heterologous species to the probe sequence is termed the "percent identity" (%ID). Therefore, a lower %ID represents greater sequence divergence between the heterologous species and *D. melanogaster *for that particular array feature. We asked to what extent statistical analysis of aCGH results recovered the actual sequence divergence between the species examined. The majority of the array features for which hybridization was significantly reduced (P < 0.1 FDR corrected) in the heterologous species relative to *D. melanogaster *are truly diverged in the heterologous species examined (Figure 1). As can be seen in Figure 1, the proportion of features identified as diverged by aCGH increased dramatically from 97 %ID to 92 %ID, with the relationship between %ID and proportion identified as diverged plateauing at either extreme. On average, 84% of the orthologs that share less than 92 %ID to *D. melanogaster *showed significantly reduced hybridization. For orthologs between 92 - 97 %ID, approximately 50% had significantly reduced hybridization, and at 97 %ID and greater an average of only 13% of features had significantly reduced hybridization. Fitting a logistic curve to these data, we estimated the limit of detectable sequence divergence as the %ID for which there is a 50% chance of a feature being called diverged by aCGH analysis (ID-50) [similar to power analysis techniques, e.g. [31]]. The ID-50 was similar for all three heterologous species (*D. sechellia*: 95.5 %ID; *D. simulans*: 94.7 %ID; *D. yakuba*: 94 %ID). It should be noted that, in general, these levels will vary depending on array quality and replication.

### Relationship between sequence divergence and hybridization ratio

The majority of aCGH studies, even in microbes, aim to identify only presence or absence of orthologs. Such studies generally employ one of two assignment strategies. The first strategy employs a cut-off threshold derived from a separate characterized strain or other published results [32,33]. The second strategy analyzes each hybridization dataset according to its intrinsic experimental variability [e.g. GACK: [34], GENCOM: [35]] in order to determine presence or absence. However, beyond a binary assignment, a more descriptive relationship between sequence divergence and hybridization ratio is possible. For example, Kim et al. [34] defined a transition zone for those genes thought to be present but highly diverged. For two genes, studied in seven microbial species, a linear relationship was found between the log hybridization ratio and percent divergence [24]. Similar studies conducted with species of the genus *Bartonella *[36] or strains of *Chlamydia *[11] also found a linear relationship between log hybridization ratio and percent sequence divergence and noted deviation from the linearity of the relationship for orthologs with < 75 %ID, of which there were few in our study.

**Figure 2:**
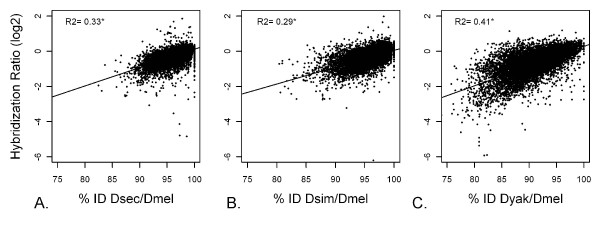
**Linear regression analysis of hybridization ratiovs. %ID**. (A) *D. sechellia*, (B) *D. simulans*, and (C) *D. yakuba *relative to *D. melanogaster*.

In order to quantify the extent to which hybridization ratio depicts the true underlying sequence divergence, we measure the correlation between these two measures. For all three species comparisons, a linear regression of %ID and log_2 _hybridization ratio showed a strong and highly significant correlation (*D. sechellia*: Multiple R^2 ^= 0.3257, P < 2.2e-16; *D. simulans*: Multiple R^2 ^= 0.2920, P < 2.2e-16; *D. yakuba*: Multiple R^2 ^= 0.4083, P < 2.2e-16) (Figure 2), with the data for *D. yakuba *showing the strongest correlation. The apparent decrease in correlations (lower R^2^) seen for *D. sechellia *and *D. simulans *compared to that for *D. yakuba *reflects the increased range of feature sequence divergence of *D. yakuba *orthologs, in addition to the contribution of technical variation rather than actual sequence variation for the less diverged *D. sechellia *and *D. simulans *orthologs.

### Sources of variation and statistical power

While technical variation in hybridization ratios exists even for within-species experiments (where sample and probe sequences are almost identical), previous work has suggested that this variation increases as sequence identity decreases [e.g. [22,37]]; however, this relationship has not been fully investigated. Here, with the incorporation of additional replicates and the consideration of variation due to hybridization kinetics, we expand our understanding of the sources of variation that cause the hybridization ratio for individual features to deviate from the value predicted by the linear regression.

**Figure 3:**
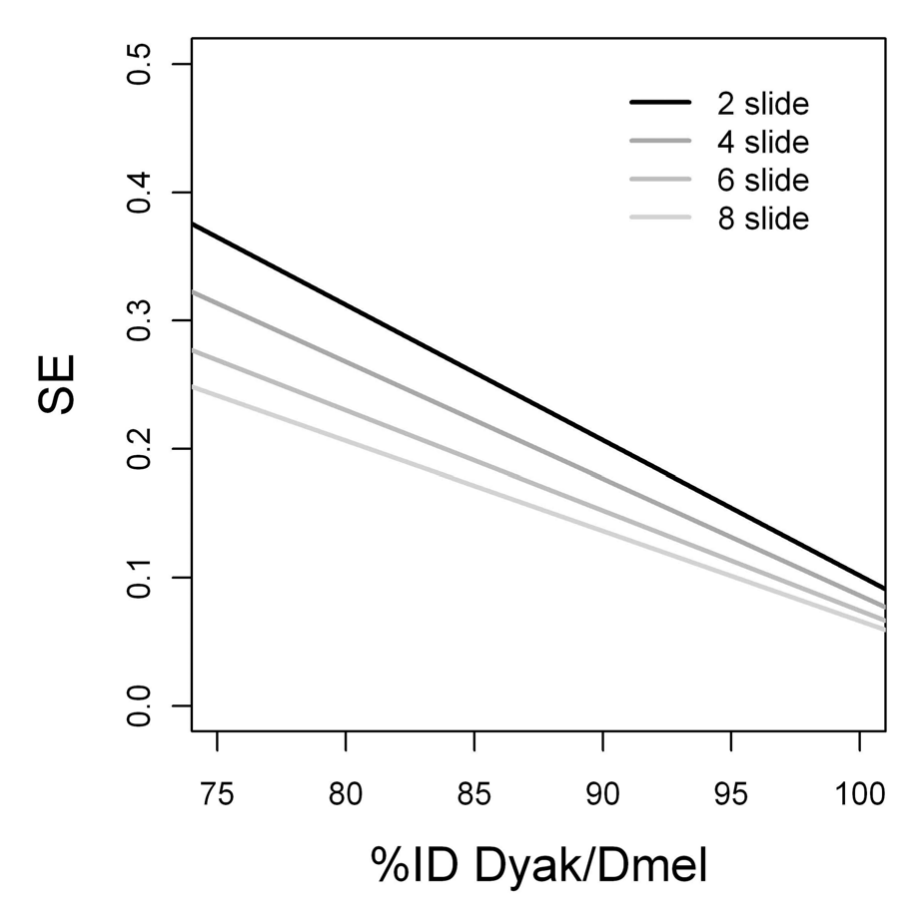
**Standard error reduction with increasing technical replication**. The standard error (SE) of the fitted value for hybridization ratio of *D. yakuba *vs. *D. melanogaster *(linear regression). The standard error decreases with increased technical replication, particularly for orthologs of lower %ID.

To test whether increased technical replication reduces technical variation, we focused on *D. yakuba*, which offers the greatest range of sequence divergence relative to *D. melanogaster*. We included six additional *D. yakuba *vs. *D. melanogaster *hybridization experiments (for a total of eight arrays) and repeated the analysis with all possible two, four, and six array combinations as well as using the full complement of eight heterologous aCGH experiments. As expected, for a given statistical threshold, increased technical replication resulted in an increase in the fraction of features that were detected as diverged and an increase in ID-50, our measure for the limit of detectable sequence divergence (see above) (Table 2). Interestingly, the R^2 ^value for the regression model of hybridization ratio on %ID was not substantially affected by the increased replication, yet the accuracy of sequence identity estimates for individual array features improved, as demonstrated by the decreased standard error of the fitted value (Figure 3). This effect was stronger for array features of 70 - 80 %ID than for features of 90 - 95 %ID. This observation has important implications for experimental design. While orthologs of greater sequence divergence are easily identified, even with few technical replicates, an accurate estimate of their sequence identity requires additional hybridizations. With eight technical replicates, an additional 5% of the variation in hybridization ratio can be attributed to %ID. The remainder of the variation (i.e. deviation from the regression line) is likely due to the individual characteristics of the sequence differences between the heterologous species and the platform species.

**Table 2:** Increased technical replication leads to an increased power to detect array features that show a difference in the hybridization strength for *D. yakuba *vs. *D. melanogaster*

# Arrays	# Analyzed	Diverged	ID-50	**R**^2^**(%ID/Hyb)**
2	15372	61%	92.8	0.4085
4	15851	75%	95.0	0.4382
6	16001	80%	95.7	0.4485
8	16060	83%	96.0	0.4530

# Analyzed: a reduced dataset due to removal of features on the X chromosome; Diverged: percent of features identified as diverged at P < 0.1 FDR; ID-50: %ID at which there is a 50% chance of a feature being identified as diverged; R^2 ^(%ID/Hyb): R^2 ^for the regression of %ID on hybridization ratio.

**Figure 4:**
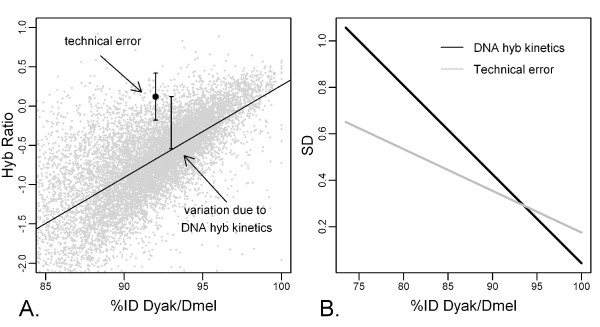
**Partitioning of variation in hybridization ratio**. Two sources of variation affect the quantitative prediction of sequence divergence from hybridization ratios. A) Schematic representation of the "technical error" (the standard error of the fit of the hybridization ratios for that feature among technical replicates) and the variation due to physical characteristics of each probe and sample DNA (DNA hybridization kinetics: the deviation from the regression line of hybridization ratio vs. %ID). B) The calculated relative contributions of technical error (grey line) and variation due to DNA hybridization kinetics (black line) as a function of %ID of *D. yakuba *to *D. melanogaster *(SD: standard deviation). At low %ID, DNA kinetics predominates.

Variation in DNA hybridization kinetics has been shown to increase with sequence divergence [22]. Such variation can be caused by any of several physical characteristics of - or differences between - sample DNA and probe DNA (including presence/absence of introns, GC content, distribution of sequence variation, length of probe, and length of sequence alignment). While minor differences between technical replicates account for the proportion of the total variation that is due to technical error, the proportion of the total variation that is due to DNA hybridization kinetics manifests as a deviation from the regression line (Figure 4A). When DNA sequence characteristics produce increased hybridization strength, the hybridization ratio for the array feature in question is expected to be greater than the value predicted by the regression model for %ID. Conversely, when DNA sequence characteristics produce decreased hybridization strength, there is an expected decrease in hybridization ratio for that array feature. Even though it was not our goal here to devise an absolute metric that accounts for all possible sources of variation, we explored the relative contribution of technical variation and variation due to hybridization kinetics.

The magnitude of the variation due to technical error is measured by the SD of the fitted value for the hybridization ratio of each feature. The magnitude of the variation due to DNA hybridization kinetics is measured by the standard deviation (SD) of the residuals from the regression line (estimated as the median absolute residual divided by 0.6745). Based on the 8-array *D. yakuba *vs. *D. melanogaster *dataset, we found that hybridization ratios for features representing conserved orthologs (greater than ~95 %ID) were more affected by technical error, whereas those of more diverged orthologs (less than ~95 %ID) were more strongly influenced by DNA hybridization kinetics (Figure 4B). Both technical error and variation due to DNA hybridization kinetics increase with greater sequence divergence.

**Figure 5:**
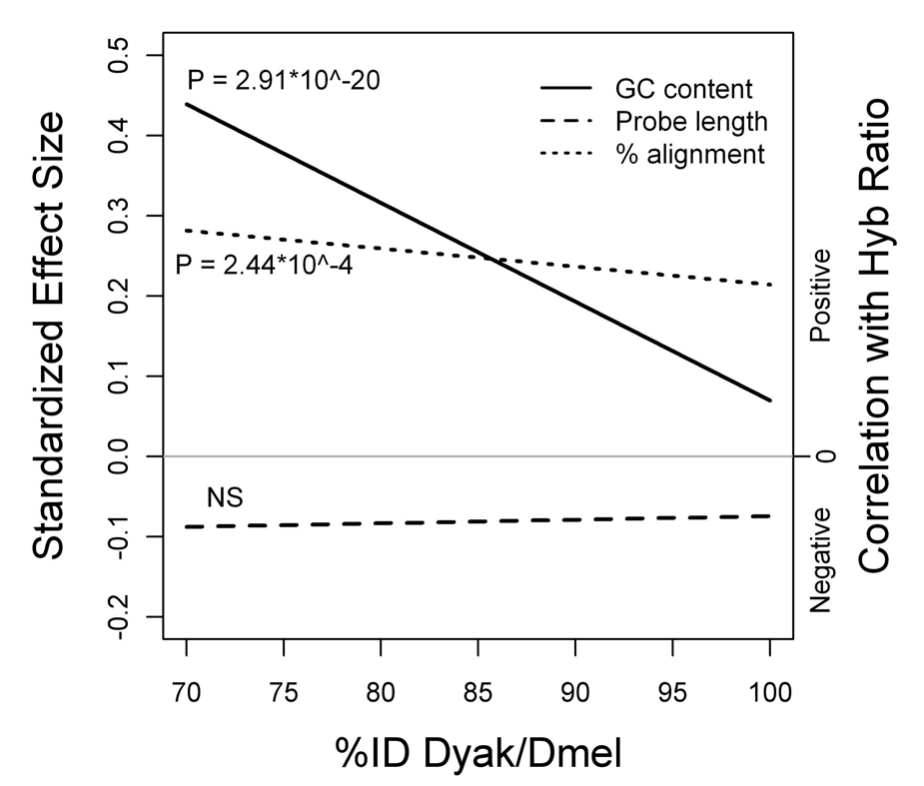
**Standardized effect sizes of probe characteristics across %ID of *D. yakuba *to *D. melanogaster***. P-values for the partial correlations of GC content, probe length, and relative alignment length of probe sequence to *D. yakuba *sequence are from the (non-standardized) regression model (Table 3). GC content and percent alignment are always positively correlated with hybridization ratio, and GC content has a significant interaction with %ID (the effect sizes decrease with increasing %ID).

To demonstrate how physical characteristics other than %ID can contribute to DNA hybridization kinetics, we considered GC content of the *D. melanogaster *probe (GC content), the length of the *D. melanogaster *probe (probe length), and the percent of the *D. melanogaster *probe length over which the heterologous sequence can be aligned (percent alignment length). We incorporated these variables into the linear regression model of hybridization ratios vs. %ID to *D. melanogaster *(hybridization ratio ~%ID * (GC + length + % align)) of the 8-array *D. yakuba *and *D. melanogaster *dataset. Both GC content and percent alignment length were significant in the model, and there was a significant interaction effect of GC content and %ID (Table 3). To assess the relative effect size for these explanatory variables, the response and explanatory variables were standardized such that the mean of each variable was 0 with a standard deviation of 1. The effect size for GC content decreased with increasing %ID, yet it retained a positive effect on hybridization ratio (Figure 5). There was also a positive effect of percent alignment length on hybridization ratio, although unaffected by %ID. At lower levels of divergence (greater than 85 %ID), percent alignment length had a greater relative effect size than GC content (Figure 5). Since high GC content is likely to produce a more stable bond between two DNA strands, it is not surprising that a higher GC content of the array probe would produce a stronger bond with a diverged sequence than would be seen at low GC content, with conserved GC regions contributing extra stability to the otherwise weak bond. It is interesting that this effect is even more pronounced at higher levels of divergence, supporting the hypothesis of stabilization of weak bonds.

**Table 3:** The regression model for %ID and sequence characteristics vs. hybridization ratio [hybridization ratio ~%ID * (%GC + length + % align))] includes the interaction of GC content, probe length, and percent alignment of *D. melanogaster *probe sequence to *D. yakuba *sequence.

	Estimate	Std. Error	t value	P value	Min	Max	1 SD
(Intercept)	-21.7825	1.1968	-18.2001	5.43E-73			
%ID	0.2018	0.0130	15.4977	1.25E-53*	73.48	100	3.67
GC	0.1538	0.0166	9.2395	2.91E-20*	123	1839	150.47
Length	-0.0005	0.0006	-0.8412	0.4002	23.77	76.19	5.29
%align	0.0309	0.0084	3.6692	2.44E-04*	6.40	118.46	8.90
%ID:GC	-0.0015	0.0002	-8.0096	1.26E-15*			
%ID:length	1.88E-06	6.49E-06	0.2902	0.7716			
%ID:%align	-0.0002	9.16E-05	-1.7257	0.0844			

Min: minimum value; Max: maximum value; 1 SD: 1 standard deviation. *significant at P < 0.05.

It is also intuitive that percent alignment length would be positively correlated with the *D. yakuba *vs. *D. melanogaster *hybridization ratio, as it is another measure of divergence that is not taken into consideration by %ID. A complete alignment indicates low divergence, whereas an incomplete alignment indicates substantial divergence. The absence of a strong correlation between relative alignment length and hybridization ratio may be due to insertions in the heterologous sequence that result in reduced hybridization strength and inflated relative alignment length (even above 100%). Our goal in discussing these variables is to demonstrate the complexity of hybridization kinetics without devising a precise metric. For that reason we did not include other possible explanatory variables, such as insertions, deletions, GC content of the heterologous sample, or even the presence of paralogs, in our discussion. These factors undoubtedly will contribute to studies in non-model species; however, without full genome sequence, little benefit would be gained from an explicit model. We see aCGH as an efficient and inexpensive method for identifying highly diverged genes among species for which little or no genomic sequence information is available. Direct sequence analysis (in multiple individuals) would be necessary to further investigate any genes of interest identified by this method (e.g., synonymous vs. non-synonymous substitutions).

### Example analysis 1: constraint within Gene Ontology categories

**Figure 6:**
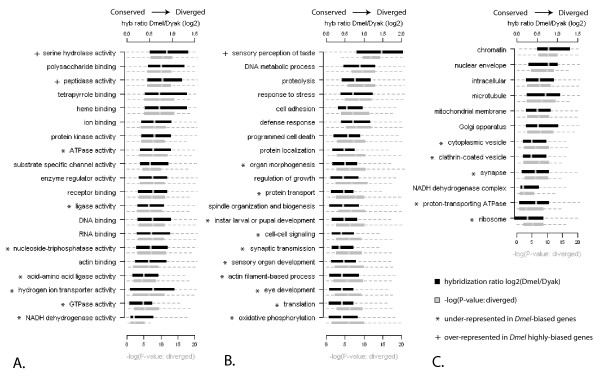
**Representative Gene Ontology statistics**. Box plots of hybridization ratio (black bar) and significance (grey bar) for *D. melanogaster*-bias in the *D. yakuba*/*D. melanogaster *8 slide analysis. The GO categories shown are selected based on number of members (greater than 10), informational content, and distribution across the ontology. Categories fall under Molecular Function (A), Biological Process (B), and Cellular Component (C).

Data from aCGH can be used to uncover trends among functional gene categories. Categories that are found to be either more diverged or conserved can provide hypotheses about which functions and pathways are under stabilizing or directional selection. Using the Gene Ontology (GO) framework, we can statistically test for over- or under-representation of specific biological processes, molecular functions, and cellular components (Figure 6). Deviations from the null hypothesis (i.e. equal representation) might suggest directional selection or evolutionary constraint.

We tested for under-representation of GO categories among the genes determined by aCGH to be diverged in *D. yakuba *(*D. melanogaster*-biased). Of the 7009 *D. melanogaster*-biased features with FlyBase gene IDs (representing 6094 genes), approximately half had GO annotations. Several GO categories were found to be under-represented, indicating increased sequence conservation and possible constraint of these processes, functions, and components in the *D. yakuba *lineage (see Additional file 1; representatives with asterisk on Figure 6). Molecular functions that were found to be under-represented in the set of diverged genes include GTPase and ATPase activities, NADH dehydrogenase activity, hydrogen ion transport activity, and acid-amino acid ligase activity as well as general ligase activity. Underrepresented biological processes include translation, ATP synthesis-coupled electron transport, oxidative phosphorylation, protein transport, actin-filament-based processes, cell-cell signaling, including synaptic transmission, and several processes associated with development (instar larval or pupal development, organ development and organ morphogenesis, eye development, and sensory organ development more generally). Finally, underrepresented cellular components include those associated with the proton-transporting ATPase, cytosol, ribosome, lipid particle, clathrin-coated vesicle, cytoplasmic vesicle, and the synapse. Overall, 111 out of 4874 GO categories that were tested (2.3%) were under-represented (i.e. more conserved) than would have been expected by chance (P < 0.01). As demonstrated in this example analysis, categories involved in very fundamental processes (ie. protein synthesis and transport, basic metabolic activity, development, and synaptic function) are likely to be more constrained.

In this example, we used GO annotations specifically for *Drosophila *genes. However, this strategy can also be applied to any non-traditional model organism (or group of species) for which there is an array platform available, based on DNA sequence similarity between array features and annotations (which are primarily derived from standard model organisms). Despite the ascertainment bias inherent to GO terms, which is largely due to the nature of hypothesis-driven research conducted in model organisms, this approach allows rigorous statistical analysis for over- and under-representation of particular molecular functions, biological processes, and cellular components [for example, see [38]]. Also, applying GO terms reduces the complexity of the data, avoids experimenter bias in cross-referencing between experiments and species, and facilitates comparisons of experimental results obtained in different organisms and/or with different platforms.

### Example analysis 2: highly diverged genes

Genes that have high divergence among species can be identified as those with substantially reduced hybridization ratios. We focus on array features with greater than four-fold reduction in hybridization ratio for *D. yakuba*. Among these 1686 highly *D. melanogaster *biased features, representing 145 genes with GO annotations, six GO categories were found to be overrepresented, including peptidase activity, serine hydrolase activity, sensory perception of taste, and three parent GO terms (P < 0.01). The enrichment for *D. melanogaster*-biased features suggests that genes in these categories are either highly diverged or are deleted in *D. yakuba*.

Among these 145 highly diverged genes with GO annotations, there were 47 that showed a four-fold reduction in hybridization strength relative to *D. melanogaster *in all three study species. This set of putatively highly diverged genes includes those with annotations to 94 different GO terms, including cell adhesion, zinc ion binding, and protein serine/threonine kinase activity (Table 4). Analysis of %ID of the array feature sequence and the top BLAST hit to the heterologous genome confirms that most of these features have a high level of sequence divergence from *D. melanogaster *in all three species or have no significant BLAST hit to the heterologous genome (e-value < 10^-14^), suggesting high sequence divergence or deletion. These data can also be analyzed according to other annotation schemes, and signatures of selection can be addressed with the cloning and sequencing of candidate genes in non-model organisms.

**Table 4:** Representative GO annotations for genes highly diverged in all test species.

FBgn	GO_ID	GO_name	Gene_name	%ID Dsec/Dmel	%ID Dsim/Dmel	%ID Dyak/Dmel
FBgn0013300	GO:0003677	DNA binding	Male-specific-transcript-35Ba	81.53	82.76	NoHit
FBgn0033015	GO:0003700	transcription factor activity	d4	NoHit	NoHit	NoHit
FBgn0036496	GO:0003729	mRNA binding	CG7804	89.8	90.52	NoHit
FBgn0039659	GO:0003729	mRNA binding	CG14506	86.81	88.06	81.48
FBgn0000303	GO:0004102	choline O-acetyltransferase activity	Choline acetyltransferase	94.38	96.3	NoHit
FBgn0051742	GO:0004175	endopeptidase activity	CG31742	93.04	92.45	89.66
FBgn0000258*	GO:0004674	protein serine/threonine kinase activity	Casein kinase II alpha subunit	90.53	89.88	87.34
FBgn0020386	GO:0004674	protein serine/threonine kinase activity	Protein kinase 61C	90.62	89.63	NoHit
FBgn0028986	GO:0004867	serine-type endopeptidase inhibitor activity	Serine protease inhibitor 3	93.21	91.91	84.96
FBgn0050289	GO:0004867	serine-type endopeptidase inhibitor activity	CG30289	NoHit	84.64	81.74
FBgn0053225	GO:0004867	serine-type endopeptidase inhibitor activity	CG33225	NoHit	84.18	NoHit
FBgn0040849	GO:0004970	ionotropic glutamate receptor activity	Ionotropic receptor 41a	NoHit	NoHit	NoHit
FBgn0050440*	GO:0005085	guanyl-nucleotide exchange factor activity	CG30440	NoHit	NoHit	NoHit
FBgn0031585	GO:0005200	structural constituent of cytoskeleton	CG2955	92.31	93.85	NoHit
FBgn0028859	GO:0005245	voltage-gated calcium channel activity	CG12455	90.96	90.4	NoHit
FBgn0037885	GO:0005515	protein binding	CG17721	88.99	89.57	NoHit
FBgn0050054	GO:0005525	GTP binding	CG30054	89.71	89.71	NoHit
FBgn0040099	GO:0005534	galactose binding	lectin-28C	87.32	87.32	82.99
FBgn0026175	GO:0006511	ubiquitin-dependent protein catabolic process	skpC	87.57	85.24	85.27
FBgn0058006*	GO:0007155	cell adhesion	CG40006	85.07	79.59	86.17
FBgn0052547	GO:0007186	G-protein coupled receptor protein signaling	CG32547	94.83	94.83	NoHit
FBgn0003373*	GO:0007594	puparial adhesion	Salivary gland secretion 3	84.15	NoHit	NoHit
FBgn0030420	GO:0008234	cysteine-type peptidase activity	CG12717	NoHit	NoHit	81.82
FBgn0031208	GO:0008234	cysteine-type peptidase activity	CG11023	83.67	83.95	83.33
FBgn0029661	GO:0008270	zinc ion binding	CG16781	NoHit	86.86	NoHit
FBgn0039498	GO:0008270	zinc ion binding	CG17991	85.2	84.31	82.4
FBgn0038005	GO:0009055	electron carrier activity	Cyp313a5	NoHit	NoHit	89.6
FBgn0013576	GO:0016998	cell wall catabolic process	l(3)82Fd	92.65	94.12	NoHit
FBgn0002855	GO:0018991	oviposition	Accessory gland-specific peptide 26Aa	84.21	83.45	NoHit

GO category shown for each gene was selected based on informational content and preponderance of evidence for that GO annotation. Genes not annotated beyond the terms molecular function, biological process, or cellular component, are not included. NoHit: no BLAST hit of array feature sequence to heterologous genome sequence (e-value < 10^-14^). *represented by multiple array features

## Conclusions

The results presented here demonstrate that aCGH can robustly detect genes that are highly diverged in a given species compared with one for which a microarray platform is available. Our use of a *D. melanogaster *microarray to estimate sequence divergence on a gene-by-gene basis for three fully sequenced heterologous species allowed for a proof-of-principle for this approach and allowed us to explore the success and biases inherent in this technique. We found a consistent and linear relationship between array hybridization ratio and sequence divergence between the sample and the platform species. The limit of detectable sequence divergence depends on the power of the experiment. While the power can be increased with additional technical replicates, there will still be a subset of diverged genes that escape detection due to other specific hybridization kinetics of the sample DNA to the array feature. This technique is generally applicable, even though thresholds, correlation strengths, and appropriate divergence distances may differ for different array platforms (cDNAs, long or short oligonucleotides). As the number of microarray platforms available for non-traditional model species is continually increasing [axolotl: [39], dolphin: [40], butterfly: [41,42], coral: [43], many fish species, for review: [44], crustaceans: [45-47]], researchers focusing on these and related model systems will continue to benefit from the relatively low cost of array hybridizations. Rapid advances in next-generation sequencing technology notwithstanding [2,48], aCGH provides an effective alternative to *de novo *sequencing of a large number of complex eukaryotic genomes.

## Methods

### Array Production

We used a *Drosophila melanogaster *microarray with ~22,000 features containing PCR products (~500 base pairs long) generated from custom primers designed to predict open reading frames [[33]; GEO platform number GPL6056]. The microarray was printed on poly-L-lysine slides (Thermo Scientific) in a 48 pin format using an OmniGrid-100 arrayer (GeneMachines). Following hydration, snap drying and UV cross-linking, the slides were blocked with succinic anhydride and sodium borate in 1-Methyl-2-Pyrrolidinone, rinsed, dried according to standard procedure [49] and stored dry until used.

### Sample Preparation and aCGH

Isogenic *Drosophila melanogaster*, *D. simulans*, *D. sechellia *and *D. yakuba *strains (Dmel\y;cn;bw;sp, Dsim\w [501], Dsec\Robertson3C, Dyak Tai18E2) were obtained from the Tuscon *Drosophila *stock center (now known as the San Diego Drosophila Species Stock Center). Genomic DNA was isolated from ~100 *Drosophila *males of each stock according to a standard ProteinaseK/Phenol:Chloroform protocol. DNA quantity and purity was assayed (via Nanodrop 1000) prior to and after DNA size reduction using a Hydroshear (Genome Solutions/Digilab) with a standard orifice set to maximal possible shearing speed (13) for 20 cycles (maximal shearing speed varies with individual orifice). This treatment resulted in fragments of 500 bp - 2 kb as determined by gel electrophoresis, visualized with ethidium bromide. Two micrograms of sheared genomic DNA was fluorescently labeled through incorporation of Cy3 or Cy5 labeled dCTP (Amersham) in a Klenow fragment (Invitrogen; Bioprime) reaction of 35 microliters according to manufacturer's protocol. Labeled sample DNA was purified by size exclusion on YM-30 filters (Eppendorf) and appropriate samples were combined. Hybridizations proceeded for ~16 hours at 65°C in a 3.4× SSC, 0.15% SDS, 1 mM DTT hybridization buffer. Male *D. melanogaster *samples were used in competitive hybridizations with two male *D. sechellia *samples, two male *D. simulans *samples and two male *D. yakuba *samples, incorporating dye swaps to account for dye bias. These aCGH hybridizations were analyzed for the ability to detect significantly diverged genes. An additional six *D. melanogaster *versus *D. yakuba *aCGH hybridizations were available in order to asses the effect of increased technical replication. For this power analysis, only genes located on the autosomes were used because a subset of the hybridizations involved *D. yakuba *female genomic DNA of the same strain.

### Microarray Data Analysis

Hybridized arrays were scanned with an Axon 4000B scanner (Axon Instruments) using Genepix 5.0 software (Axon Instruments). All raw array data have been submitted to GEO database (dataseries number GSE18416 sample number GSM459056-67). Features of poor quality (signal intensity < 2 standard deviations above background) and those of potentially erroneous sequences (mismatch between initial PCR product sequence prediction and current *D. melanogaster *database; refseq_rna 12/2008) were excluded. Features were only considered in the analysis if they survived these technical filters on multiple arrays for a given species comparison. Raw data from Genepix was imported into R, and LIMMA [50] was used to apply a background correction ("minimum") and within-array intensity normalization ("loess"). Because we expect the normalization of cross-species arrays to be affected by a substantial number of diverged genes in the non-platform species [51], we performed the within-array normalization using a set of ~1000 genes highly conserved (greater than ~95% sequence identity; determined with NCBI BLAST to Genbank) among *D. melanogaster D. simulans *and *D. yakuba*. A linear model was fitted to the data using "lmFit", and "eBayes" provided error shrinkage towards a pooled estimate of variation [52]. Array features were tested for hybridization ratios that were significantly different from equal as assessed after a FDR multiple testing correction at P < 0.1 [53]. The Gene Ontology analysis was conducted in GOstats [54], using features with FlyBase IDs, as listed in the GEO platform.

### Genomic Sequence Divergence

The sequences of the *D. melanogaster *probes were predicted by blasting primers from the *D. melanogaster *probe (GEO Profiles accession: GPL6056) against the *D. melanogaster *Release 5 assembly and searching for unique and proximal (within 600 base pairs of each other) targets. We queried the resulting sequences against the *D. simulans *and *D. yakuba *NCBI genomes (chromosome) using "megablast" (2009) and against the full chromosome sequence assemblies for *D. sechellia *downloaded from flybase.org (release 1.3). From each heterologous genome, the top BLAST hit to each array feature (threshold e-value 10^-14^) was used to obtain the percent similarity between the two sequences and the length of the alignment.

## Authors' contributions

SR and RK conceived of the study. HM and AJ performed the statistical analysis. KS carried out the microarray hybridizations. RK, HH, AJ, SR and HM participated in the design of the study and prepared the manuscript. All authors read and approved the final manuscript.

## Supplementary Material

Additional file 1 **Statistical analysis of Gene Ontology term representation**. Gene Ontology terms, and uncorrected p-value, found to be over- and under-represented according to a hypergeometric test, among the set of genes found to diverged (significantly *D. melanogaster*-biased) in the *D. yakuba*/*D. melanogaster *8-slide analysis). Click here for file
